# Impact of an antibiotic stewardship programme in a surgical setting

**DOI:** 10.4102/sajid.v36i1.307

**Published:** 2021-11-24

**Authors:** Muhammad A. Bashar, Jacqui Miot, Evan Shoul, Robyn L. van Zyl

**Affiliations:** 1 Pharmacology Division, Department of Pharmacy and Pharmacology, Faculty of Health Sciences, University of the Witwatersrand, Johannesburg, South Africa; 2 Department of Pharmacology and Therapeutics, College of Health Sciences, Federal University Birnin Kebbi, Birnin Kebb, Nigeria; 3 Department of Internal Medicine, Faculty of Health Sciences, University of the Witwatersrand, Johannesburg, South Africa

**Keywords:** antibiotic, antibiotic resistance, antibiotic stewardship, surgery, South Africa, antibiotic prophylaxis, surgical site infection, antibiotic usage

## Abstract

**Background:**

Antibiotics are miracles of science and critical for many surgical procedures. However, the emergence of multidrug resistant pathogens resulting from inappropriate antibiotic use is a threat to modern medicine. This study aimed to determine the appropriateness of antibiotic use, cost, consumption and impact of an antibiotic stewardship intervention round in a surgical ward setting.

**Methods:**

Baseline antibiotic utilisation was determined with a retrospective cross-sectional study in two surgical wards in a tertiary academic hospital in South Africa where medical records of 264 patients who received antibiotics were reviewed. In the second stage of the study, records of 212 patients who received antibiotics were reviewed during a weekly antibiotic stewardship intervention round. The volume of antibiotics consumed was determined using defined daily doses (DDDs)/1000 patients’ days, and the appropriateness of the antibiotic prescription for treatment was also determined using a quality-of-use algorithm.

**Results:**

There was a reduction in the volume of antibiotic consumption from a total 739.30 DDDs/1000 to 564.93 DDDs/1000 patient days, with reduction in inappropriate antibiotic use from 35% to 26% from baseline to antibiotic stewardship programme stages, respectively. There was an overall increase in culture targeted therapy in both wards in the antibiotic stewardship programme stage.

**Conclusion:**

The implementation of an antibiotic stewardship programme led to a reduction in antibiotic consumption and improvement in appropriate use of antibiotics.

## Introduction

Antibiotics are a precious resource whose discovery has transformed modern medicine by playing a critical role in the fight against infectious diseases and decreasing mortality caused by bacterial infections.^1,2^ The rapid development of resistance to available antibiotics by bacteria, and lack of development of new agents over the years has negatively affected this initial success.^2^ Antibiotic resistance (ABR) is a global threat to both public health and economic stability, especially in developing countries. A United Kingdom (UK) study predicted that if left unchecked, ABR will claim an extra 10 million lives annually causing the global economy a loss of over US$ 100 trillion by 2050.^3^ Hence, the urgent need for implementation of successful antibiotic stewardship programmes (ASP) to slow down resistance to antibiotics and conserve last line treatments.

Many surgical conditions like diverticulitis, appendicitis and cholecystitis are infectious in nature, and often treated with antibiotics,^4^ therefore, surgeons need to use antibiotics prudently. Despite advancements in prevention and control of infections, surgical site infections (SSIs) have remained leading cause for mortality and morbidity.^5^ Patients with SSIs are more likely to be readmitted with a higher risk of death, than those without these infections, they also require longer hospitalisation and incur considerable increases in healthcare costs.^5^ Optimising antimicrobial use before, during or after surgical procedures is critical in addressing ABR, simultaneously reducing the burden of infection globally.^6^ This requires a systematic approach by way of antibiotic stewardship to optimise rational antibiotic use. A limited number of studies have been conducted to determine the appropriateness of antibiotic therapy in surgical settings, especially in developing countries.^7^ Hence, the need to involve surgeons in ASPs. An Egyptian study demonstrated that engaging with surgeons with interests in rational antibiotic prescribing to educate their colleagues, can improve optimal antibiotic use for surgical prophylaxis in hospitals.^8^

Antimicrobial stewardship has been described as:^9^

[*C*]oordinated interventions designed to improve and measure the appropriate use of antimicrobial agents by promoting the selection of the optimal antimicrobial drug regimen including dosing, duration of therapy, and route of administration. (p. 323)

An ASP involves several strategies with different approaches, which when properly implemented will improve patient outomes.^9^ The main aim of ASPs are to ensure that patients receive appropriate antibiotic therapy, improve clinical response, with reduced adverse outcomes.^10^ Antibiotic resistance is a direct function of antibiotic use, as the volume of antibiotics consumed increases, so do the chances of developing resistance by bacteria as a survival mechanism.^11^ Over-consumption of antibiotics is a major driver of ABR,^12,13,14^ therefore, appropriate consumption in an ASP helps improve management of antibiotic utilisation.^15^ Apart from reducing the emergence of resistance, ASPs are also associated with reductions in drug acquisition cost, toxicities and infections caused by pathogenic bacteria such as *Clostridioides difficile*.^16^ To help address the effects of ABR and optimise antibiotic use, the South African Antibiotic Stewardship Programme (SAASP) has published a pocket guide to help prescribers.^17^

A survey to assess the level of implementation of ASP across continents has shown that in Africa only 14% of hospitals had such programmes in place, compared to 53% in Asia.^18^ An initial implementation of ASPs in South African public and private hospitals led to a reduction of antibiotic consumption,^19,20,21^ and also resulted in antibiotic cost reduction.^19,20^ However, they were implemented in medical ward settings and did not specifically address ASPs in surgical wards. This study aims to determine the appropriateness of antibiotic use, cost, consumption and impact of an antibiotic stewardship round in a surgical ward setting.

## Methods

### Setting

Charlotte Maxeke Johannesburg Academic Hospital is a tertiary academic teaching hospital of the University of the Witwatersrand, providing a wide range of specialist care and serves as a referral hospital for many hospitals in the Gauteng province and all over Africa. The study was conducted in the vascular (395) and general/gastroenterology (396) surgical wards of this hospital.

### Study design

The study was conducted in two stages as follows.

#### Baseline stage

This was a retrospective, cross-sectional medical record review study of patients admitted and who received antibiotics (*n* = 264) in the study wards from February 2016 to May 2016. The variables assessed included: age, gender, diagnosis, indication for surgery, types of antibiotic used, indication, dosage, route of administration, allergy, nature of specimen collected for culture and sensitivity, and biomarker results. These biomarkers included: procalcitonin and C-reactive protein to guide and monitor antibiotic therapy. Results from the National Health Laboratory Services (NHLS) database were used to complement pathology results obtained from patient records. The total volume of antibiotics consumed was determined using defined daily doses (DDDs)/1000 patient days.^22,23^ The number of DDDs for each antibiotic was determined by converting the total amount of that antibiotic used over a given duration into grams and the result was divided by the standard World Health Organization (WHO) DDD value of that antibiotic in grams. The appropriateness of antibiotics used for therapeutic purposes was determined using a guideline developed by Gyssens et al.^24^ The appropriateness of surgical prophylaxis was determined based on the recommendations of the SAASP,^17^ and Essential Medicines Lists for South Africa.^25^ Each prescription was compared to what should be the best practice according to these guidelines and assessed against the Gyssens’ categories to determine whether they were appropriate or not (Table 1). The choice of prophylactic agent, dose and duration of prophylaxis was assessed, however the time of the administration of first dose before first surgical incision was not assessed.

**TABLE 1:** Gyssen’s algorithms for evaluation of the appropriateness of antimicrobial drug therapy.

Category	Reason categorisation
I	Agree with the use of antimicrobial therapy/ prophylaxis, the prescription is definitely appropriate
II	The antimicrobial drug therapy/prophylaxis prescription is inappropriate due to:
IIa	1. improper dosage
IIb	2. improper dosage interval
IIc	3. improper route
III	The antimicrobial drug therapy/prophylaxis prescription is inappropriate due to:
IIIa	1. excessive length
IIIb	2. duration too short
IV	The antimicrobial drug therapy/prophylaxis prescription is inappropriate due to:
IVa	1. more effective alternative agent (Aa): specify
IVb	2. less toxic Aa: specify
IVc	3. less expensive Aa: specify
IVd	4. less broad spectrum Aa: specify
V	The antimicrobial drug therapy/prophylaxis prescription is unjustified: use of any antimicrobial is not indicated
VI	Records insufficient for categorisation.

Gyssens IC, Van Den Broek PJ, Kullberg B-J, Hekster YA, Van Der Meer JW. Optimizing antimicrobial therapy. A method for antimicrobial drug use and evaluation. J Antimicrob Chemother. 1992;30(5):724–727. https://doi.org/10.1093/jac/30.5.724

#### Antibiotic stewardship programme stage

The ASP stage was conducted from June to September 2016, and involved a dedicated ASP weekly round in both study wards. This was separate from the daily routine round conducted by the surgeons. The antibiotic stewardship round was led by an infectious diseases specialist with a team consisting of a pharmacist, a MSc in Clinical Pharmacology from the University Pharmacology Division with the support of a clinical microbiologist. A pre-antibiotic round was conducted by the lead investigator the day before the main round to retrieve results of laboratory investigations and obtain consent from patients to participate in the study. On the day of the ASP ward round, each condition was discussed at the bedside especially regarding antibiotic selection and laboratory investigations with emphasis on collection of appropriate specimens for cultures. Fellows, residents and interns in surgery also participated in the rounds. The ASP interventions also involved early conversion from intravenous to oral agents, dose optimisation and adjustment of dose in patients with renal and hepatic impairment. Patients who were on antibiotics were all reviewed during the rounds, however only consented patients’ records were included in the data analysis. All enrolled patients were followed for the duration of their admission stay to ensure compliance of the recommendations made during antibiotic rounds. The same types of variables were collected as in the baseline stage. The appropriateness of antibiotic prescriptions, cost and volume of antibiotic consumed were determined using the same criteria as in the first stage.

### Ethical considerations

The study was approved by the University of the Witwatersrand Human Research Ethics Committee with an approval number: M151142. All participants gave their informed consent for inclusion before they participated in the study.

### Statistical analysis

Data was analysed using Stata software version 14 (StataCorp, College Station, TX, USA). An independent sample *t*-test was used to compare the differences in volume of antibiotic consumption between the two stages. There were 17 and 12 patient records in the baseline and ASP stages, respectively, that were incomplete. The results were mostly related to culture and biomarkers, and they were excluded in the analysis.

## Results

The results of each stage are presented but not compared because of differences in patient populations between the two stages. There were more male patients in both stages of the study as shown in Table 2. The baseline stage of the patients in the baseline stage was 51.50 ± 15.91 years, while in the ASP stage it was 45.77 ± 16.81 years (*p* = 0.01). The average length of hospital stays (LOS) was significantly increased in the ASP stage (*p* = 0.01). In the baseline stage, amoxicillin/clavulanic acid was the most frequently used antibiotic (53%), followed by piperacillin/tazobactam (16%); while in the ASP stage, although still the most used agent, the frequency of amoxicillin/clavulanic reduced to 34%, while piperacillin/tazobactam increased to 26% (Table 2).

**TABLE 2:** Patient demographics and clinical indicators.

Variables	Baseline stage (%)	ASP stage (%)	*p*
Demographics	-	-	-
Number of patients	264	212	-
**Gender**			0.501
Male	150 (56.82)	114 (53.77)	-
Female	114 (43.18)	98 (46.23)	-
Age (years) Mean + s.d.	45.77 ± 16.81	51.50 ± 15.91	0.01
LOS (days)	8.33 ± 7.58	13.44 ± 11.83	0.01
DoT for therapeutic purposes (days)	4.74 ± 4.58	3.96 ± 2.04	0.01
Antibiotic consumption (DDD/1000 patient days)	739.30	564.93	0.038
Total number of antibiotic prescriptions (percentage of inappropriate antibiotic used therapeutically)	443 (35)	442 (26)	0.006
Total prescriptions administered IV	396 (89.39)	372 (84.16)	0.341
Appropriately prescribed IV antibiotics	252 (56.90)	269 (60.80)	0.302

ASP, antibiotic stewardship programmes; s.d., standard deviation, LOS, length of hospital stay; DoT, duration of antibiotic therapy;DDDs, defined daily doses; IV, intravenous.

*p* values calculated using the chi-square test or *t*-test where appropriate.

More specimens were collected for culture in the ASP stage. A total of 397 and 491 cultures were requested in the baseline and ASP stages, respectively. The collection of appropriate specimens such as tissue instead of superficial swab was emphasised in the rounds. The collection of tissue specimens for culture was more in the ASP stage (28.8%) compared to the baseline stage (15.3%). The request of superficial swabs for culture in the vascular ward reduced from 22.7% in the baseline stage to 11.2% in the ASP stage.

The intravenous route of administration was the most utilised route in both stages, where 89% and 84% of all drugs were administered intravenously in the baseline stage and the ASP stage, respectively. There was a higher number of culture targeted therapies prescribed in both wards in the ASP stage compared to the baseline stage (see Appendix 1). Macrolides such as azithromycin were used as prokinetic agents to stimulate gastrointestinal motility in newly operated patients. There was a reduction in the use of antibiotics for empirical therapy in the general ward during the ASP stage.

In the baseline stage, 443 antibiotic prescriptions were ordered for therapeutic purposes, of which 35% were inappropriate based on the algorithm used, whilst in the ASP stage, 442 antibiotic prescriptions were ordered of which 26% were inappropriate. In both stages, the main reasons for inappropriate prescriptions were the use of more broad-spectrum (Gyssens’ category IVd) and more expensive agents (Gyssens’ category IVc), where narrow spectrum and less expensive agents were available. Overall, 64% and 59% of the antibiotic use for prophylactic indications in the baseline and ASP stages, respectively, was inappropriate (Table 3). Amoxicillin/clavulanic acid was the most commonly used agent (68%) for surgical prophylaxis in the baseline stage followed by cefazolin (29%). According to the classification system used, based on the South African guidelines, nearly 65% of all surgical prophylaxis was inappropriate based on the agent choice, while 7.3% was inappropriate based on duration of prophylaxis. Similarly, in the ASP stage, amoxicillin/clavulanic acid and cefazolin were the two most frequently used agents for surgical prophylaxis, and 62% of all surgical prophylaxis in this stage was inappropriate based on choice of agent; with 6.6% inappropriate based on the duration of prophylaxis. Piperacillin-tazobactam was given at 4.5 g qid while cefazolin was administered at the dose of either 1 g or 2 g tds. in both wards. Up to 71% of the recommendations made during the antibiotic stewardship rounds were implemented. Reasons were not given on why some of the recommendations were not implemented.

**TABLE 3:** Appropriateness of peri-operative antibiotic prophylaxis.

Variables	Baseline stage (%)	ASP stage (%)
Overall inappropriate use	64	59
Inappropriateness due to agent choice	65	62
Inappropriateness due to duration more than 24 h	7.3	6.6
Time of the administration of the first dose	Not assessed	Not assessed

ASP, antibiotic stewardship programmes.

**FIGURE 1:**
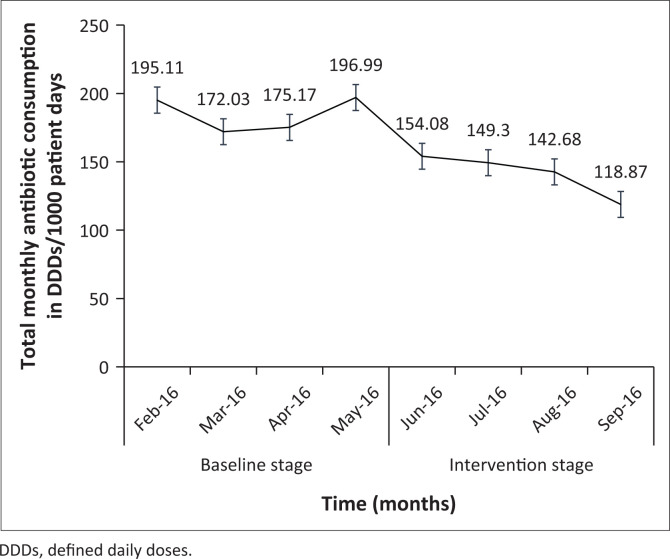
Trend of antibiotic consumption over the study period.

A reduction (24%) in the total volume of antibiotic consumption was observed after implementation of the antibiotic stewardship round by 174.37 DDD/1000 patient days. The trend of the total monthly antibiotic consumption in the two stages is shown in Figure 1. There was an increase in the utilisation of piperacillin/tazobactam in the ASP stage (Table 4), which was because of an increase in the prevalence of *Pseudomonas aeruginosa* compared to the baseline stage (Appendix 2). Consumption of the five most commonly used agents in both stages of the study is presented based on clinical indications in Appendix 3.

**TABLE 4:** Frequency of top 10 antibiotic prescribed over a 10-month period in the general and vascular surgical wards.

Name of antibiotics	Number of prescriptions (%) at baseline stage		Number of prescriptions (%) at ASP stage		Difference in number (%) of prescriptions issued	
	*n*	%	*n*	%	*n*	%
Amoxicillin/clavulanic acid	233	52.59	149	33.71	↑ 84	18.88
Piperacillin/tazobactam	72	16.26	115	26.02	↓ –43	−9.76
Cefazolin	36	8.13	31	7.21	↑ 5	0.92
Metronidazole	20	4.52	18	4.07	↑ 2	0.45
Fluconazole	13	2.93	19	4.29	↓ –6	−1.36
Vancomycin	10	2.26	6	1.39	↑ 4	0.87
Cloxacillin	7	1.58	3	0.68	↑ 4	0.90
Imipenem	7	1.58	8	1.81	↓ –1	−0.23
Ciprofloxacin	6	1.35	13	2.72	↓ –7	−1.37
Ertapenem	2	0.45	18	4.07	↓ –16	−3.62
Others	37	8.35	62	14.03	↓ –25	−5.68
Total	443	100.00	442	100.00	-	-

ASP, antibiotic stewardship programmes.

## Discussions

This ASP intervention study was conducted in a surgery setting of a South African academic hospital with an antibiotic stewardship team led by an infectious diseases specialist. Over-consumption and inappropriate use of antibiotics are major drivers of ABR with consequent additional hospital costs.^11^ Inappropriate use of antibiotics includes use of antibiotics at suboptimal doses, incorrect route of administration or poor adherence to prescribed drugs.^26^ It also involves the use of antibiotics without a defined bacterial/fungal infection or an indication for prophylaxis,^26^ and the use of last resort agents, while there are effective first and second line antibiotics.^27^

A baseline stage was used to describe the current standard of antibiotic usage in each of the wards. Both the baseline and ASP ward round targeted all patients on antibiotics. A wide range of surgical cases were admitted in the general ward. The cases included obstructed hernias, intestinal obstruction and cutaneous abscesses, perforated peptic ulcer diseases, and oesophageal carcinomas. Others are: lower gastrointestinal bleeding, rectal carcinomas, acute cholangitis, cholecystitis, pyogenic liver abscesses, primary liver cell carcinomas, peri-pancreatic sepsis, appendicitis, and overwhelming post-splenectomy sepsis. In the vascular ward, the cases included acute and chronic vascular ischemic diseases, diabetic foot diseases, stab injuries, and many other vascular conditions.

The demographic characteristics of the patients show that more male patients were admitted to the surgical wards in both stages of the study, which is mirrored in other studies conducted in other countries.^28,29^ The patients in the ASP stage had a higher mean age compared to those in the baseline stage which may have clinical implications in that older patients may have a higher risk of having other comorbidities, complications or severity of disease. Previous studies in surgical settings conducted in Malaysia and Jordan reported average ages of between 43 and 51 years,^6,30^ respectively, which is similar to those reported in this study. Although many ASPs have reported a reduction in LOS during interventions,^22,31^ in contrast, this study showed an increase in LOS. The average LOS of patients in the baseline and ASP stages were 8.33 ± 7.58 and 13.44 ± 11.83 days, respectively. This may be because of the fact that this study was conducted in a surgical setting and older patients were admitted during the ASP stage compared to baseline stage. Another reason for an increased LOS in the ASP stage was an increase in the prevalence of *Pseudomonas aeruginosa* in the ASP stage, which was statistically significant (*p* = 0.01) compared to the baseline stage. Despite an increase in LOS in this study, there was a reduction in antibiotic consumption and duration of antibiotic therapy (DoT) in the ASP stage. During ASP rounds, it was found that many patients (especially those with ischemic vascular conditions) stayed longer on admission waiting for surgical procedures. As most of them did not have infections, it was unnecessary for them to be on antibiotics so these were stopped during ward rounds. The number of laboratory tests per patient may be driven by an increased length of stay where more tests are ordered with a longer LOS.

Globally there is no standard metric for quantifying antibiotic consumption across all clinical settings,^32^ and the WHO has recommended the use of DDDs,^18^ defined as ‘the average maintenance dose of the drug when used for its primary indication in adults’.^33^ Expressing antibiotic consumption by DDDs/1000 patient days enables comparison between hospitals^33^ and clinical settings, as was implemented in this study. A key finding in this study was lower antibiotic consumption in the ASP stage. The ASP programmes in South African public and private hospitals have also reported reductions in antibiotic consumption,^19,20,21^ as well as in developed countries.^23,34,35^

The use of antibiotics when there is no indication for their use, such as to treat clean wounds and using it for prolonged duration or failure to de-escalate to narrow spectrum when the results of culture are available, are considered inappropriate. To encourage appropriate antibiotic use it was emphasised during rounds that antibiotics should only be used empirically when there is clear indication, and effort should be made to collect appropriate specimens for culture before starting empiric treatment and should be reviewed when the culture results are available. There were discussions during the rounds on what is considered appropriate such as prescribing antibiotics at the right dose via the right route of delivery. It was previously reported that both public and private Intensive Care Units (ICUs) across South Africa had a high percentage of inappropriate use of antibiotics of 44% and 61% in public and private ICUs, respectively.^36^ This is higher than what is observed in the current study (26% – 35%), possibly because those studies were conducted in the ICU setting where higher antibiotic consumption is more likely. Studies in other developing countries such as India and Malaysia have also shown a high percentage (39% – 42%) of inappropriate antibiotic usage.^7,29^ However, this is not limited to developing countries, a Swiss study using Gyssens’ algorithm found a total of 32% of all prescriptions to be inappropriate which was high (37%) in patients who received antibiotics as treatment compared to those who received it for prophylaxis (17%).^37^ In the Netherlands, 16% – 29% of all antibiotic prescriptions were judged inappropriate using Gyssens’ algorithm, and this was mostly because of unjustified prescriptions.^38,39^

Surgical prophylaxis is an area of concern with respect to appropriate antibiotic usage. A high proportion of inappropriate antibiotic utilisation was seen in prophylactic usage in the baseline (65%) and ASP (62%) stages of this study, based on the wrong choice of agents, while 7.3% and 6.6% were inappropriate based on a duration of more than the recommended 24 h, respectively. Of the antibiotics prescribed, amoxicillin/clavulanic acid was the most frequently used prophylactic agent in both stages. The use of amoxicillin/clavulanic acid is not recommended for surgical prophylaxis by the SAASP and Standard Treatment Guidelines and Essential Medicines Lists for South Africa for most of the procedures conducted on patients at the time of this study.^17,25^ Augmentin and cefazolin are prescribed prophylactically in theatre at doses of 1.2 g bd and 1 g tds, respectively, but are then often continued unnecessarily for days on the wards.

Inappropriate use of prophylaxis has been found to be even higher in surgical settings of other developing countries; a Malaysian study found 66% of prophylaxis to be inappropriate, with 35% because of inappropriate duration and 16% as a result of wrong choice.^7^ A study in a Jordanian cardiac surgery centre, found that up to 98% of surgical prophylaxis was inappropriate based on wrong antibiotic choice, while 59% was inappropriate with a duration of more than 48 hr as recommended by the guideline.^30^ A study in a private surgical setting in India found that 32% of surgical prophylaxis was inappropriate because of choice of agents, while 37% was inappropriate on account of prolonged duration of prophylaxis.^40^ Appropriate surgical prophylaxis reduces the risk of potential contamination during surgery, thus lowering the chance of SSIs.^41^ On the other hand, inappropriate surgical prophylaxis is associated with drug-related adverse events and *Clostridioides difficile* infections at the individual level, and increases the risk of developing resistant pathogens and healthcare costs at the community level.^42^

A lower mean DoT for therapeutic purposes was found in the ASP stage of 3.96 ± 2.04 days compared to 4.74 ± 4.58 days in the baseline stage. A short DoT with an antibiotic is recommended by the World Society of Emergency Surgery in the management of intra-abdominal infections.^43^ A short DoT reduces the risk of developing resistance, adverse events and cost, and is recommended in all patients except in situations where there is difficulty in achieving source control, sepsis, and in immunocompromised individuals.

The programme was well accepted in the wards with no resistance from the surgeons or ward staff. During the programme, a 71% compliance rate to the recommendations was recorded and although this is a favourable outcome considering the short duration of the study, it is lower than that reported in other studies.^44,45^ The compliance rate may have been better if surgical consultants participated more actively in the rounds. In this study, it was found that Gyssens’ algorithm is a useful and reliable tool in determining appropriateness of antibiotic prescriptions and can be recommended to be used in future studies. In the authors’ opinion, the incorporation of antibiotic stewardship rotations and inclusion of short courses on appropriate antibiotic use in the management of surgical infections in the curriculum of South African surgery-related residency programmes will go a long way in ensuring optimal rational antibiotic usage and reducing the burden of SSIs. These results have clinical implications in that the level of inappropriate antibiotic usage for surgical prophylaxis in baseline and ASP is high, mostly because of incorrect agents being prescribed and for a prolonged duration of prophylaxis. This may increase rates of resistance, chances of acquiring SSIs and additional costs to the patients. The high rate of inappropriate surgical prophylaxis in the ASP stage was most likely on account of the majority of antibiotics being started in the operating theatre, whereas this intervention was conducted in the wards. Possible measures to improve appropriate antibiotic use for surgical prophylaxis could include adherence to national guidelines and educational programmes targeting theatre staff and surgeons on rational antibiotic usage.

## Study limitations

A limitation of this study is that it was conducted at two different time points and therefore it was not possible to determine if there was a significant impact of the ASP ward rounds on inappropriate antibiotic usage. Another limitation is that it was conducted in only two surgical wards of an academic hospital and this makes it difficult to generalise the findings. It may be problematic to replicate this study in many non-teaching hospitals in South Africa and across the African continent, because of the shortage of ASP experts outside of the academic setting. The Netcare model in which non-specialist pharmacists successfully drove the ASP in a South African group of private hospitals is an alternative option.^21^ Although the ASP ward round in this study showed a lower volume of antibiotic consumption and inappropriate antibiotic use compared to the baseline stage, which are important drivers of resistance, it was not possible to say this would lead to a reduction in ABR, because the study was conducted over a short period of time. Data collection was challenging because of lack of electronic patient records as well as monitoring the implementation of recommendations made during the antibiotic stewardship rounds.

## Conclusion

Introduction of a weekly ASP round in two surgical wards of a tertiary South African hospital showed a lower level of antibiotic consumption, improvement in the appropriate use of antibiotics and a reduction in the duration of antibiotic therapy. Hospitals in developing countries are encouraged to optimise the use of antibiotics by implementing ASP programmes.

## Appendix 1

**TABLE 1-A1:** Antibiotic therapy based on clinical indications.

Clinical indications	Baseline stage number of prescription (%)						ASP stage number of prescription (%)					
	Vascular ward		General ward		Total of both wards		Vascular ward		General ward		Total of both wards	
	*n*	%	*n*	%	*n*	%	*n*	%	*n*	%	*n*	%
Empiric	58	56.31	186	54.71	244	55.08	82	56.94	158	53.02	240	54.30
Prophylaxis	32	31.07	95	27.94	127	28.67	37	25.69	60	20.13	97	21.95
Targeted	12	11.65	55	16.18	67	15.12	25	17.37	63	21.15	88	19.90
Prokinetic	1	0.97	4	1.17	5	1.13	0	0.00	17	5.70	17	3.85

**Total**	**103**	**100.00**	**340**	**100.00**	**443**	**100.00**	**144**	**100.00**	**298**	**100.00**	**442**	**100.00**

ASP, antibiotic stewardship programmes.

## Appendix 2

**TABLE 1-A2:** Pathogens isolated in the two stages of the study.

Cultured pathogens	Baseline stage	ASP stage	*p*
*Escherichia coli*	21	22	0.46
*Pseudomonas aeruginosa*	18	29	0.01
*Klebsiella pneumoniae*	17	23	0.27
Methicillin-sensitive *Staphylococcus aureus*	12	5	0.001
*Enterococcus cloacae*	1	11	0.001
*Proteus mirabilis*	11	5	0.01
*Enterococcus faecium*	7	15	0.01
*Clostridium difficile*	5	8	0.032
Coagulase-negative *Staphylococcus*	5	13	0.01
Methicillin-resistant *Staphylococcus aureus*	4	8	0.02
*Streptococcus agalactae*	4	2	-
*Acinetobacter baumannii*	15	15	-
*Enterococcus faecalis*	9	9	-
*Burkholderia cephacia*	-	3	-
*Citrobacter koseri*	-	3	-
*Staphylococcus epidermidis*	4	1	-
*Morganella morganii*	3	-	-
*Acinetobacter iwoffii*	-	2	-
*Bacteroides eggerthi*	-	2	-
*Serratia marcescens*	-	2	-
*Streptococcus gallolyticus*	-	2	-
*Trischosporon mucoides*	-	2	-
*Micrococcus species*	-	1	-
*Bacteroides thetaiotaomicron*	-	1	-
*Citrobacter braakii*	-	1	-
*Citrobacter freudii*	-	1	-
*Streptococcus mitis*	-	1	-
*Pseudomonas fluorescen*	-	1	-
*Haemophilus influenzae*	-	1	-
*Prevotella oralis*	-	1	-
*Achromobacter xylosoxidans*	-	1	-
*Stenotrophomonas maltophilia*	-	1	-
*Fusobacterium necrophorum*	-	1	-
*Gemella morbillorum*	-	1	-
*Candida albicans*	3	8	-
*Enterobacter aerugenes*	3	2	-
*Candida glabrata*	2	1	-
*Bacillus specie*	2	-	-
*Candida parapsilosis*	2	-	-
*Streptococcus constellatus*	2	-	-
*Providentia rettgeri*	2	-	-
*Streptococcus anginosus*	1	1	-
*Streptococcus haemolyticus*	1	-	-
*Corynebacterium species*	1	1	-
*Prevotella bivia*	1	-	-
*Peptostreptococcus anaerobius*	1		-
*Clostridium perfringes*	1	-	-
*Klebsiella oxytoca*	1	1	-
*Micrococcus specie*	1	-	-
*Trichosporon asashi*	1	-	-

**Total**	**160**	**208**	**-**

ASP, antibiotic stewardship programmes.

## Appendix 3

**TABLE 1-A3:** Consumption of most frequently used agents.

Most used antibiotics	Indication		
	Prophylaxis (95% CI)	Empiric (95% CI)	Targeted (95% CI)
**DDD per 1000 patient days in the baseline stage**			
Amoxicillin/clavulanic acid	28.14 (21.66–34.63)	170.69 (147.14–194.24)	28.26 (19.21–37.31)
Piperacillin/tazobactam	-	147.57 (61.86–233.29)	22.91 (15.07–30.74)
Fluconazole	52.08	83.14 (49.14–117.15)	-
Metronidazole	0.15	19.46 (7.35–31.57)	3.87 (1.73–6.00)
Cefazolin	7.99 (6.75–9.24)	-	-
**DDD per 100 0 patient days in the ASP stage**			
Amoxicillin/clavulanic acid	12.41 (8.45–16.38)	107.05 (89.76–124.33)	14.05 (7.12–20.98)
Piperacillin/tazobactam	-	90.16 (79.30–100.93)	27.68 (21.54–33.83)
Fluconazole	12.13 (9.32–14.93)	79.53 (47.52–111.54)	23.54 (2.49–44.58)
Ertapenem	-	5.35 (3.58–7.11)	21.04 (13.97–28.11)
Cefazolin	11.06 (6.25–15.87)	-	-

ASP, antibiotic stewardship programmes; CI, confidence interval, DDD, defined daily doses.
